# Comparative In Situ Study of Dynamic Load Generated by Gravel Piles Measured by a Fiber-Optic Interferometer

**DOI:** 10.3390/s22155579

**Published:** 2022-07-26

**Authors:** Martin Stolárik, Stanislav Kepák, Miroslav Pinka, Jakub Čubík, Jan Nedoma

**Affiliations:** 1 Department of Geotechnics and Underground Engineering, Faculty of Civil Engineering, VSB—Technical University of Ostrava, 708 00 Ostrava, Czech Republic; martin.stolarik@vsb.cz (M.S.); miroslav.pinka@vsb.cz (M.P.); 2 Department of Telecommunications, Faculty of Electrical Engineering and Computer Science, VSB—Technical University of Ostrava, 708 00 Ostrava, Czech Republic; jakub.cubik@vsb.cz (J.Č.); jan.nedoma@vsb.cz (J.N.)

**Keywords:** vibration measurement, gravel piles, anthropogenic sources, seismic monitoring, civil engineering, fiber-optic sensor, interferometer

## Abstract

Currently, all the technology used for seismic monitoring is based on sensors in the electrical domain. There are, however, other physical principles that may enable and fully replace existing devices in the future. This paper introduces one of these approaches, namely the field of fiber optics, which has great potential to be fully applied in the field of vibration measurement. The proposed solution uses a Michelson fiber-optic interferometer designed without polarization fading and with an operationally passive demodulation technique using three mutually phase-shifted optical outputs. Standard instrumentation commonly used in the field of seismic monitoring in geotechnical engineering was used as a reference. Comparative measurements were carried out during the implementation of gravel piles, which represents a significant source of vibration. For the correlation of the data obtained, the linear dependence previously verified in laboratory measurements was used. The presented results show that the correlation is also highly favorable (correlation coefficient in excess of 0.9) from the values measured in situ, with an average deviation for the oscillation velocity amplitude of the optical sensor not exceeding 0.0052.

## 1. Introduction

Seismic monitoring is an integral part of the implementation of most geotechnical structures where vibration-generating equipment is used. These vibrations can adversely affect both the surrounding buildings in terms of adverse dynamic effects on the construction of building structures, which is dealt with in technical standards at the national or European level, as well as the comfort of the population, when the generated vibrations have a negative effect on the human body, which is covered by sanitary and government regulations.

From the point of view of technical seismicity acting on buildings, we monitor the maximum amplitudes of velocity or acceleration obtained on the basis of in situ seismic measurements in the building and the prevailing frequencies. The magnitude of the amplitude and frequency pattern depend on the source of the vibration, the distance of the object being monitored, and the geological environment through which the vibration propagates through the foundation joint and foundation structure to the upper structure. As the distance from the source of dynamic effects increases, the intensity of these vibrations decreases, so one of the basic tasks of seismic engineering is also to determine the attenuation characteristics of the rock environment.

An important source of vibration in geotechnical engineering is mainly equipment designed to modify the properties of the rock environment, such as vibratory packers, vibratory plates, and vibratory rollers, which accelerate the primary consolidation process through vibration, as well as deep foundation technologies in the form of drilled, driven, or vibrated piles. In the case of these technologies, we are usually talking about harmonic oscillations with a predominant frequency determined by the manufacturer of the equipment used and with oscillation speed amplitudes in the range of tenths to tens of millimeters per second.

For seismic monitoring, i.e., monitoring the effects of technical seismicity, standard seismic instrumentation consisting of a three-component velocity or acceleration sensor operating on the electrical principle and a digital seismic station is used. This measurement system has been used for several decades, but in the developing 21st century, other physical principles are also becoming available that could successfully complement or even replace the established standard with a number of benefits. In the case of successful implementation in seismic engineering, several advantages can be found, be it the design of the sensor itself, resistance to external influences, and even price.

The results of previous experimental studies dealing with the use of different physical principles in the monitoring of the effects of engineering seismicity have confirmed the applicability of the alternative devices under development, especially in the frequency domain. The logical follow-up was to focus on the time domain, where a first (laboratory) experiment performed in a homogeneous environment with a calibrated strike yielded interesting and thought-provoking results [1]. This served as the impetus for a larger-scale follow-up experiment, in which vibrations generated during the implementation of in situ gravel piles were monitored using standard seismic instrumentation, which acted as a reference in the experiment, and by an optical interferometric sensor under development.

The article presents the entire experiment carried out on the construction of a road bypass in the Czech Republic, the results of this experiment with emphasis on correlations in the time domain, and the subsequent determination of the attenuation characteristics of the rock environment using data obtained from the alternative monitoring device under development.

The fundamental innovation of the presented solution is mainly in applications in the field of anthropogenic vibrations. The research on the following showed basic experimental applications in the field of natural seismicity and vibrations around high-speed railways and tunnels, without comparison to a reference gauge.

## 2. State of the Art

In this part of the article, the problem of seismic monitoring of vibrations from anthropogenic sources using commercially manufactured seismic instrumentation for this purpose is presented, as well as an alternative approach in the form of a physical principle based on optical fibers which can be used for vibration measurement. In conclusion, the actual implementation of the gravel piles whose dynamic effects were monitored is presented.

### 2.1. Commercially Manufactured Seismic Instrumentation

Three-component commercially manufactured seismic stations operating in the electrical domain are commonly used throughout the world in a wide range of applications, be it the monitoring of natural seismicity, induced seismicity, or engineering seismicity due to anthropogenic activity. In the field of technical seismicity, which is dealt with by the authors, there are two major areas of interest.

The first is the area of blasting, both in the excavation of underground works, especially shallow below the surface and in the intracity of large agglomerations [2], and also in the extraction of minerals in surface quarries by blasting, where these quarries are often located close to civil buildings, especially in Central Europe [3]. In these cases, the results of seismic monitoring are used to optimize the blasting operations: limit and total charge, as well as charge timing, in addition to monitoring the impact of the blasting or mining on structures.

The second major area is the monitoring of dynamic expressions of heavy motor vehicles, especially rail transport, which have an undesirable effect on structures [4], or the monitoring of dynamic phenomena related to technological processes on construction sites, especially in the area of improving foundation conditions by compaction or the implementation of deep foundations [5]. In all these cases, seismic stations are usually installed as solitary temporary meters located in buildings according to applicable national legislation and standards.

With the help of profile measurements, when using multiple seismic stations, it is then possible to derive the attenuation of a given environment from the measured values and to predict the range of dynamic loading in the vicinity of the vibration source based on the attenuation curves. Last but not least, seismic measurements are also used to calibrate advanced mathematical models.

The common frequency range of seismic stations used is from 2 to 200 Hz, and the nature of the time domain recording depends on the particular source of dynamic loading. The fundamental disadvantage of these devices is the mechanical principle of the measurement itself, which makes the measuring instrumentation highly sensitive to handling. In contrast to newly proposed fiber-optic sensor technology, these commonly used devices are also not immune to electromagnetic influences and cannot withstand extreme climatic conditions in the long term.

### 2.2. Optical Sensors

The authors are building on the results of previous work. Papers [6,7] focus on monitoring vibrations caused by blasting or other sources of dynamic loading. This uses fiber-optic sensors in combination with conventional vibration measurement equipment. As a result, the pilot studies provide a good starting point for the further deployment of these alternative devices. Other achievements include the measurement of vibration during landform compaction with a vibratory roller [8], the study of harmonic vibration in comparative measurements of alternative equipment and instrumentation for seismic monitoring [9], and research on vibration associated with works and construction technologies in brownfield areas [10]. Last but not least, the authors presented a description of new methods for seismic monitoring, namely a laboratory comparison of fiber-optic sensors with pneumatic measurement systems [1].

Vibration measurement with a focus on seismic monitoring (earthquakes, blasting, mine-induced seismicity, construction work, etc.) can be approached in various ways, one of which is fiber optic technology. These are classified according to the application, principle, technology, or design of the sensor itself. For example, a description of these technologies is discussed in [11].

Nowadays, many publications mainly focus on distributed measurements using optical fibers. A specific example is the distributed acoustic sensor (DAS), which evaluates the Rayleigh scattering coming back from a transmitting device. This way, it can turn a single-mode fiber into several thousand acoustic-vibration sensors. This system has been described and used by the authors of papers [12,13]. Distributed measurements using optical fibers are usually implemented on fibers for telecommunication purposes, but their cladding and mechanical robustness can be optimized [14]. The field of measurements with DAS is diverse: it can be, for example, the study of Earth tide frequencies for the definition of bedrock properties [15] or systems for seismic monitoring [16].

Another fiber-optic technology presented in the field of vibration measurement is fiber Bragg gratings (FBG). This is an inscribed periodic structure that changes the refractive index directly into the optical fiber. This structure can change the reflected wavelength on the mechanical stresses applied to it. This principle can be encountered, for example, in [17], where a three-dimensional accelerometer consisting of Bragg grating structures embedded in a seven-core optical fiber is presented. The technology of Bragg grates as seismometers, their production, and application are being addressed by various teams of authors, for example, the authors of publications [18,19]. They are analyzing their fiber-optic grating seismometers and improving their accuracy and fabrication methods [20,21]. The FBG measurement system is used in various applications, such as seismic activity monitoring in coal mines [22], or health structure monitoring [23]. The most scientifically active areas of FBG sensors are evaluation methods and their encapsulation [24,25].

The latest fiber-optic system technology suitable for seismic monitoring is phase sensors. Specifically, these are fiber-optic interferometers that evaluate the phase change in light between the measuring arm and the reference arm. These sensors can come in a variety of configurations and have one common denominator: high sensitivity to vibration.

The first reports come from 2014, when the authors [26] present a fiber-optic Michelson interferometer-based accelerometer. However, the solution only describes the encapsulation of the measuring arm of the interferometer, with the optical fiber forming the outer winding around the transducer and it has only been verified in the laboratory. The sensor design was taken further by the authors of the publication [27]. The solution describes the housing of the entire sensor head, which now includes both the measuring and reference arms of the Michelson interferometer forming the fiber-optic accelerometer. Numerical simulation of the natural frequency with the result of 258.38 Hz had been carried out using the finite element method (FEM). Sensor performance was evaluated in the laboratory on a shaking table against a reference accelerometer. According to the authors, the sensor is potentially suitable for ocean floor seismic event monitoring; however, no experiments outside the laboratory were presented. For practical use, the encapsulation of the coupling element, whose fibers up to the sensor head are sensitive to vibrations and thus effectively form part of the sensor, will have to be solved.

The three-dimensional interferometric system is used by the authors of publications [28,29], for example, to monitor seismic activity on the seabed. This type of sensor can be embedded in multicore fibers for compactness [30,31]. This sensor can be used, for example, as an accelerometer to measure low-frequency vibrations [32,33]. The Michelson fiber-optic interferometer as a seismic sensor can also be found in articles regarding various applications, such as the vibration measurement of oil wells [34] or general use in seismic applications [35,36,37,38]. In [35], a prototype device for seismological applications was developed. In this case, the light source is fed into the device via optical fiber, but the measurement system is assembled using bulk optics. The measurement of vibration disturbances in a subway tunnel during construction work is the subject of this article [37]. It is a proof-of-concept technology without in-depth analysis of measured data and frequency analysis. The authors of the paper [38] developed a system to measure non-anthropogenic sources of vibration in three directions, who installed their equipment in the seismic observation cave at the Earthquake Administration Bureau.

Three-component commercially manufactured seismic stations operating in the electrical domain are commonly used throughout the world in a wide range of applications, be it the monitoring of natural seismicity, induced seismicity, or engineering seismicity due to anthropogenic activity. In the field of technical seismicity, which is dealt with by the authors, there are two major areas of interest.

### 2.3. Gravel Piles

There are two methods of installing gravel piles in the soil. The first is by means of a vibrating or vibroflotation device, and the second method is pre-driving by so-called Franki piles. During installation, vibrations are mainly generated and transmitted through the rock environment [39].

Gravel piles are used to improve the mechanical and physical parameters of cohesive and non-cohesive soils. For example, they can be used to improve the deformation properties of soils in existing road embankments and therefore limit the settlement of any new embankments intended for road widening [40]. Another application can be to optimize the transfer of stress from the superstructure to the soil. With the use of gravel piles, smaller foundation dimensions can be used [41] and the construction will have a lower economic and environmental impact. Gravel piles can also be used to modify the strength properties of the soil environment and thus may be used, for example, to stabilize potentially dangerous slopes [42].

The most typical use of gravel piles, however, is under new embankments to limit consolidation while strengthening the subgrade. The consolidation limitation is implemented by shortening the drainage path, which is reflected in the design of the pile grid [43]. For this purpose, gravel piles were also installed at the site where the experimental measurements presented in this article were carried out.

## 3. Methods

This section of the paper focuses on the specific instrumentation used to measure the dynamic response of the subsurface from the effects of hammer-drop impact during the implementation of in situ gravel piles, both commercial seismic equipment and the fiber-optic system under development.

### 3.1. Compact Seismic Station BRS32

**Figure 1:**
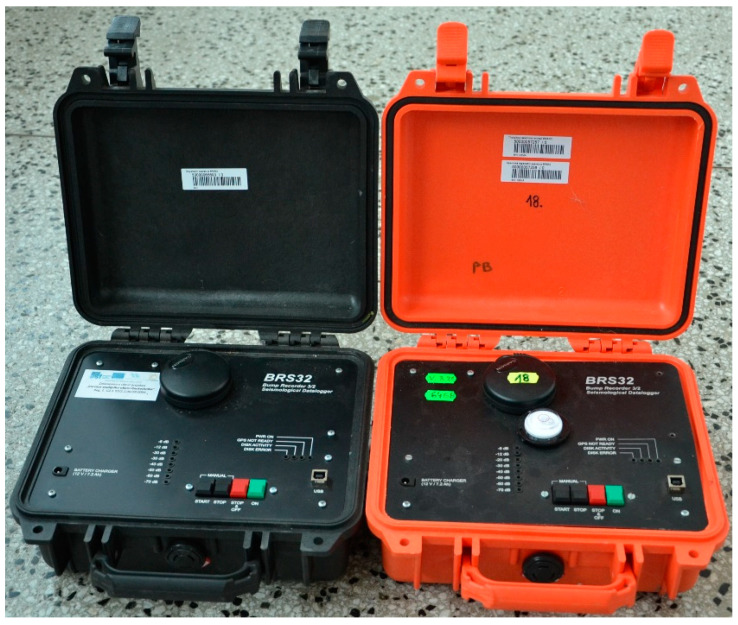
BRS32 seismic stations used as reference meters.

Two BRS32 seismic stations, see Figure 1, were used as the reference instrument at two sites: these had also been used by the authors in previous studies aimed at comparing results obtained from alternative approaches to vibration measurement [1,6,8]. The special mobile compact datalogger had originally been developed for the Institute of Rock Structure and Mechanics of the CAS of the Czech Academy of Sciences, v.v.i. and was subsequently put into custom production in the Czech Republic. This station is mainly used in the Visegrad Group countries for all seismic engineering tasks, especially in the fields of monitoring of technical and induced seismicity, and also in geophysics [44,45,46].

The instrument contains three 31-bit A/D converters that allow the sampling of input signals in the frequency range of 250 Hz to 4 kHz and the storage of the measured data, together with data from a GPS receiver (time and position), which is part of the instrument, on a built-in flash drive with a capacity of up to 64 GB. A trio of SM6-3D seismometers with a frequency range of 4.5 to 100 Hz is housed inside the instrument as standard, but the sensors can also be connected externally. For data transfer, the device offers a high-speed USB interface and, when connected to a computer, it acts similar to a regular USB drive. The entire seismic station is built into a rugged PeliCASE case that allows the station to be completely buried in the ground. The instrument is powered by an internal battery with a running time of up to 40 h, or from an external source for long-term seismic monitoring.

### 3.2. Fiber-Optic Measurement System under Development

Fiber-optic sensing uses the physical properties of light as it travels along a fiber for a measurement of quantities. One of the physical properties of light is a phase. In an optical fiber, changes in external conditions (e.g., vibration) change the optical path length and thus the phase delay of the light passing through the fiber. The phase delay *ϕ* can be described by the following term [47],

(1) $$\phi =\frac{2\pi }{\lambda }nL=\beta L,$$

where *λ* is the wavelength in a vacuum, *n* is the fiber core refractive index, *L* is the fiber length and *β* is the propagation coefficient of the fiber. The vibrations result in a change in the core Δ*n* and cladding refractive indices of the fiber due to photoelastic effect, its length Δ*L* due to strain effect and, to a lesser extent, the fiber core diameter change Δ*d* due to Poisson effect. The phase change ∆*ϕ* generated in the fiber can be then written as [48]:

(2) $$\Delta \phi =\left(\Delta \beta \right)L+\beta \left(\Delta L\right)=L\frac{\partial \beta }{\partial n}\Delta n+L\frac{\partial \beta }{\partial d}\Delta d+\beta L\frac{\Delta L}{L},$$

where *d* is the core diameter. The elasticity of the fiber can be expressed by the Young’s modulus *Y* and the Poisson ratio *ν* representing the transverse expansion (contraction) coefficient. According to the direction of the forces applied to the optical fiber, we distinguish axial strain, lateral radial and lateral unidirectional pressure. When a force is applied to a section of fiber in its axial direction, it will be stretched or compressed and an axial strain occurs. The lateral radial pressure applies the same force to the optical fiber from all directions, so the axial mechanical strains are the same for all directions. The fiber section under radial pressure is much longer than its transverse size, so the axial stress here can be neglected, and the axial strain can be deduced from the transverse stress. Under this condition, stresses and strains only exist in the x–y plane. Under lateral unidirectional pressure, which is the case of the sensor under development, the stress and strain distributions are usually analyzed in a circular disk model, compressed by two equal and opposite pressures applied at the ends of a fiber core diameter. Eventually, (2) can be rewritten as [48]:

(3) $$\Delta \phi =\beta L\frac{P}{Y}\left(1-2\nu \right)∙\left[\frac{1}{2n^{2}\left(p_{11}+2p_{12}\right)}-1\right],$$

where *p*_11_ and *p*_12_ are photoelastic coefficients of the optical fiber and the resulting phase shift is proportional to the external pressure *P* applied. At a distance, all waves approach plane wave propagation, and the relationship between pressure and particle velocity is directly proportional (this is particularly valid for the sound waves). When transverse waves (typically vibrations) propagate in a medium, pressure remains constant in the direction of wave propagation, but the particles are displaced perpendicular to the direction of the wave propagation, and with that the pressure, which is then recorded by our sensor in the z-axis.

Interferometers are then used to measure the phase shift between the light wave traveling through the sensor (or so-called measuring arm) and a reference wave split from the same radiation source. There are several possible configurations of the fiber-optic two-beam interferometer, and Michelson is one of the suitable configurations for vibration measurement.

A fiber-optic coupler and a pair of mirrors form a Michelson interferometer. Between the coupler and the mirror, there is a measurement path and a reference path, respectively. For directional coupling and decoupling of light, a circulator or isolator is used. A photodetector is connected to the output, while the input is connected to the radiation source.

**Figure 2:**
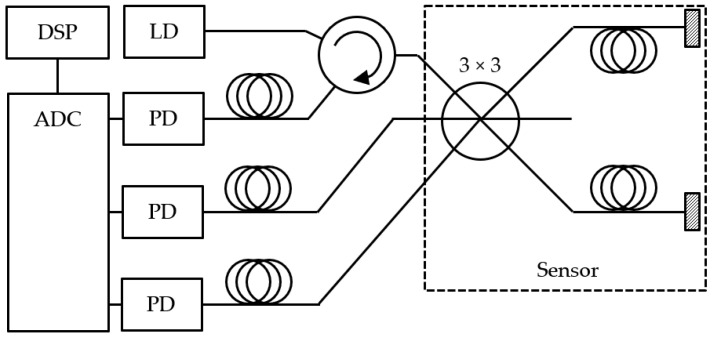
Proposed system connection diagram.

The proposed system connection diagram of the fiber-optic measurement system is in Figure 2. The radiation source is the distributed feedback laser diode (LD) operating at a wavelength of 1550 nm and with an output power of 3 mW. A three-port circulator is used for directional coupling. The optoelectronic part is further composed of three pieces of photodetectors (PD) with p-i-n diodes made with InGaAs. The ADC used was the NI-9222 module in the cDAQ-9171 chassis made by National Instruments. Digital Signal Processing (DSP) and demodulation were performed on a laptop containing our custom-developed LabVIEW application.

Interrogation of conventional two-beam interferometers is necessary as the phase shift is wrapped in the following term [46]:

(4) I(t)=C+Acos[Δϕ(t)],

where *C* is the mean value of the optical intensity, *A* is the amplitude of the variation of the optical intensity and ∆*ϕ*(*t*) is the phase shift between the measurement and the reference path.

A demodulation technique is essential to unwrap the phase shift and acquire the sensor data. Several different methods can be used for this purpose, one of which is homodyne demodulation [49]. The passive variant works on the principle of symmetry of the 3 × 3 coupler having three mutually phase-shifted outputs [47]:

(5) $$u_{n}=C_{n}+A_{n}\cos\left[\Delta \phi \left(t\right)+\frac{2\pi n}{3}+\delta_{n}\left(t\right)\right],$$

where *n* = 1,2,3 and *δ_n_* is 3 × 3 coupler phase asymmetry. Before the demodulation, it is necessary to equalize signal so the *C* and *A* values for all channels are *C_n_* = 0 and *A_n_* = 1. The phase change can be then easily unwrapped using a mathematical combination of two or three signals produced by the 3 × 3 coupler, in this case [50]:

(6) $$\Delta \phi \left(t\right)=\mathrm{arc}\tan\left[\frac{\sqrt{3}\left(u_{2}-u_{3}\right)}{u_{2}+u_{3}-2u_{1}}\right].$$

The phase shift measurement and dynamic range are given by the photodetector bandwidth and the sampling rate of the ADC used.

**Figure 3:**
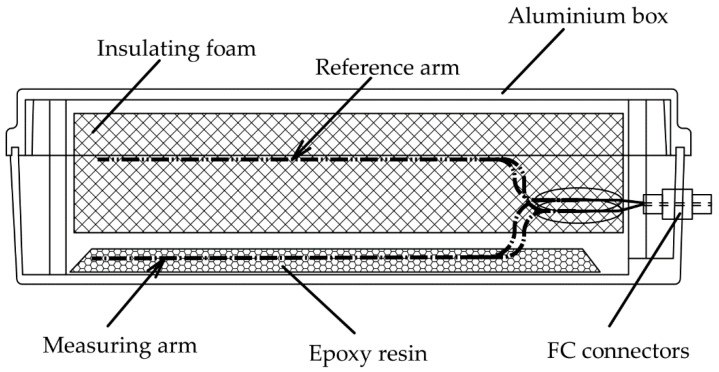
Sensor internal layout.

The sensor consists of a 3 × 3 coupler, and one pair of fiber mirrors with a fiber length of 3 m. The layout of the internal sensor can be seen in Figure 3. The outer case of the sensor consists of a waterproof aluminum box with dimensions 253 × 159 × 72 mm. A measuring arm in the form of a fiber mirror was attached to the bottom of the box using epoxy resin. A reference arm of the same optical length (within the coherence length of the LD used) was loosely placed in vibroacoustic insulating foam to minimize the transmission of vibrations to this arm. Optical FC connectors were built into the front panel that represent the input and output ports of the sensor. The sensor was connected to the evaluation site by a 100 m-long multi-fiber-optic cable.

The test measurements of a vibration test bench found that in the limited frequency range of 5–50 Hz given by the bench used (Netter NEG 5020), the observed sensor response was almost linear (statistically insignificant difference). The dominant frequency of the hammer impact lies in this frequency range and therefore the following amplitude–frequency analysis was not affected by the frequency dependence of the sensor sensitivity.

## 4. Setup of Experimental Measurement

The experimental measurements were carried out during the construction of the structure SO101 as part of the construction of the road 1/11—northern bypass of Opava (49.9562050N, 17.9048656E). The structure will consist of a transition embankment between two bridges that span the Ostrá stream and the local road 0468 towards Poland. During the design of the embankment, unsatisfactory properties of the underlying soil layers were discovered during the exploratory works. One of the biggest problems was the presence of groundwater from a nearby watercourse. As a result, different types of improvement of rock environment properties were proposed. One of the main measures included the construction of a set of gravel piles to strengthen the underlying soil and, at the same time, accelerate the process of consolidation of the underlying layers.

**Figure 4:**
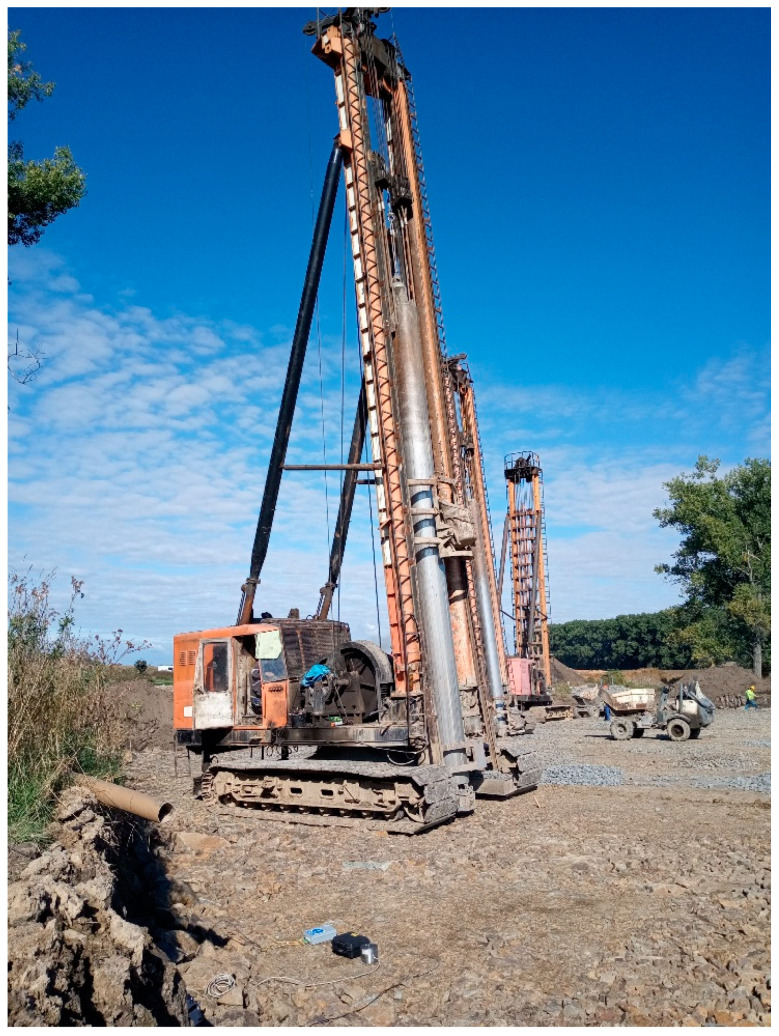
Measurement site (red site A)—on the left optical sensor under development, in the middle BRS32.

Gravel piles are made of crushed or quarried aggregate. They are installed in mixed and fine-grained soils. The installation was carried out with a heavy crawler (Figure 4) and consisted of the drilling of a borehole in the soil, which was then filled with aggregate. The borehole is protected by a steel casing. The outer diameter of the casing is 520 mm. The total length of the borehole was 12 m. The internal void space of the pile is filled with loose aggregate. The aggregate is continuously forced into the soil surroundings, while the casing is simultaneously pulled up. Forcing is performed with a drop hammer. An aggregate of approximately 1.5 m^3^ is always added, and this is then forced and compacted by a 3.5 t drop hammer falling from a height of 8 m. With each blow, the aggregate is forced into the surrounding area to form a gravel pile with a minimum diameter of 600 mm. The shock generated by the drop hammer was the subject of the experimental measurements.

The experimental measurement consisted of measuring the dynamic effects of the impact of the drop hammer on the surface layers of the soil. Measurements were carried out at two sites. The entire procedure of the installation of the gravel pile was monitored by continuous measurement. The installation of a pile consisted of the phases of arrival of the machine at a predetermined site, drilling to the required depth and then piling the aggregate and compacting it with a drop hammer. From the evaluation of the entire record, the phase with the largest amplitude was determined to be the hammer impact phase, which was an order of magnitude greater than the other phases described above. As a result, this phase was identified for evaluation and processing. Two machines were installing the gravel piles on the site. One was always performing the installation, while the other was moving to another station. There was also normal construction activity by the construction company at the site, but despite this seismic noise, the dynamic effect of the hammer strike was several times greater and therefore clearly identifiable.

**Figure A1:**
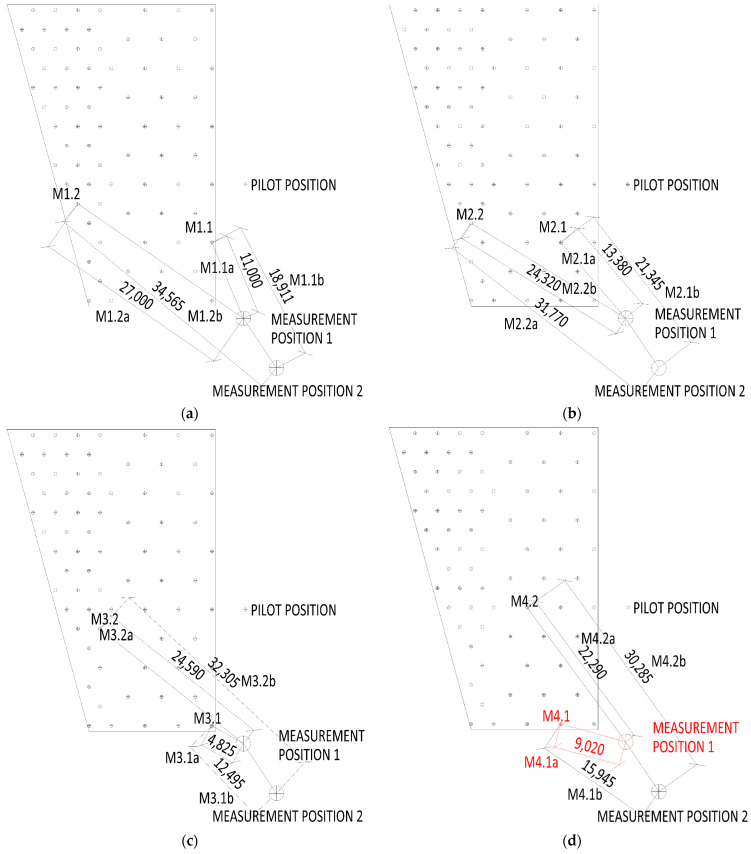

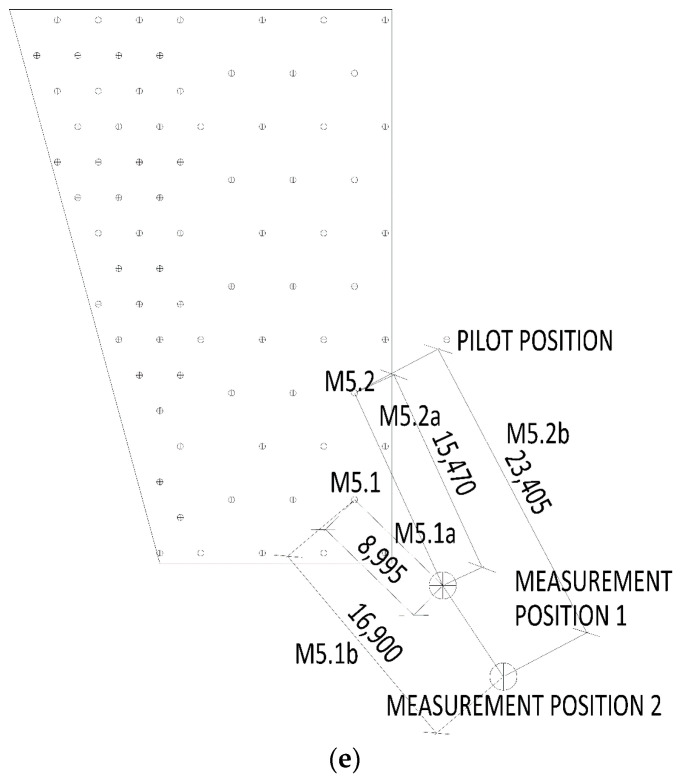
(**a**–**e**) Measuring situations 1–5 with distances between gravel piles and measuring stations marked.

Figure A1a–e shows a schematic of the individual measurements. The measurements of a gravel pile installation were always performed at two sites (site “a”; site “b”). It was measured in five situations. In one situation, two gravel pile installations were always measured. Each installation was performed with a different machine of the same type. For this paper, situations where individual piles (pile 1; pile 2) were installed one after the other and their effects which could be clearly distinguished were always selected. However, there were also cases of simultaneous installation during the measurements, but these were discarded due to ambiguous interpretation. Figure A1 shows the distances (in mm) between the gravel pile installation being monitored and the site for each situation.

For the sake of clarity, each distance is described by a name in the Mx.ya/b format. When M indicates a measurement, the first number indicates the situation, the second number indicates the pile’s serial number (1 or 2) and the letter (a or b) indicates if the distance to “a” or “b” is indicated. The example marking M4.1a (whose waveforms are shown in figures below) indicates a measurement at the fourth situation of the first pile at position “a” with a distance of 9 m (this measurement configuration is marked red, see Figure A1d).

## 5. Results

The following section presents the results of the experimental measurements in both the time and frequency domains from both the commercially produced BRS32 seismic station and the fiber-optic system under development.

**Figure 5:**
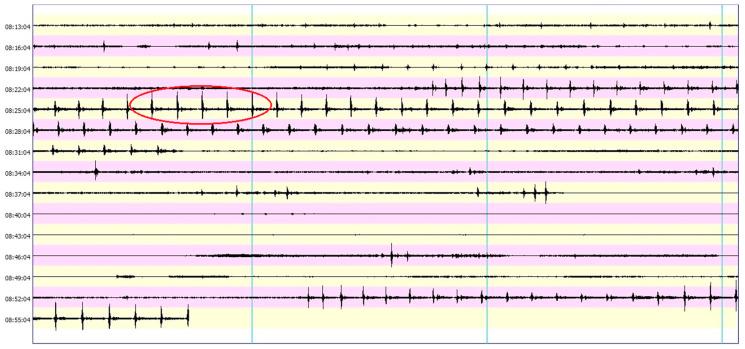
Sample file—commercial seismic station BRS32, five seismic events marked in red.

For further processing, only those data were selected that corresponded to the pre-driving phase in the Franki pile process, and the highest vibrations are generated in this phase. The set of five consecutive most powerful blows of the hammer was always chosen. An example of a data set from the BRS32 seismic station, directly from the BRSmonitor seismic data processing software, with five marked seismic events is shown in Figure 5.

**Figure 6:**
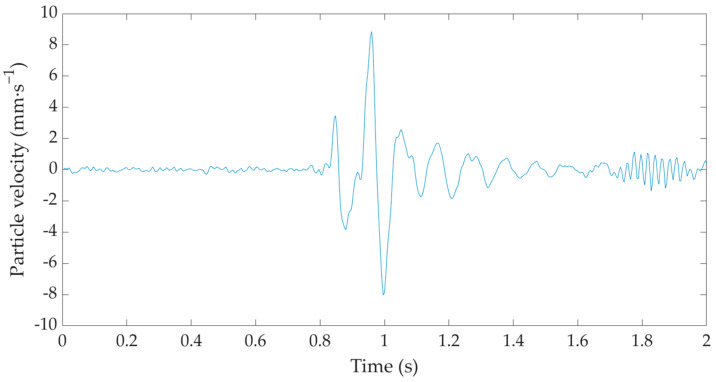
Time record of a hammer strike during the implementation of the gravel pile from the seismic station BRS32—M4.1a measurement diagram.

Figure 6 shows an example of a recording of a hammer strike during the implementation of a gravel pile obtained from the measurement of the seismic station BRS32 at a distance of 9 m from the implemented pile—measurement diagram M4.1a (see Figure A1d). The horizontal axis represents the GPS time and the vertical axis represents the amplitude of the oscillation velocity in mm·s^−1^.

The BRS32 works as a three-component seismometer that measures in three mutually perpendicular axes: vertical, horizontal radial and horizontal transverse. Due to the fact that the experimentally developed sensor was omnidirectional and registered the prevailing direction of the oscillation, the data from the BRS32 station were only evaluated in the vertical direction, which corresponded to the direction of the hammer strike. In the near zone where the measurements were performed, almost identical values of the oscillation velocity were registered for all three components. The nature of the recording corresponds to a typical manifestation of an isolated dynamic phenomenon, where there is a rapid and abrupt increase in the peak and a very rapid decay.

**Figure 7:**
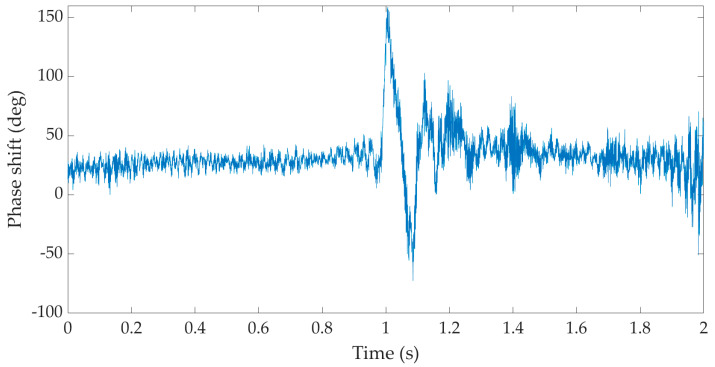
Time recording of one hammer strike during the implementation of a gravel pile from the fiber-optic system under development—measurement diagram M4.1a.

Figure 7 is an example of a recording of one hammer strike during the implementation of a gravel pile obtained on the basis of optical interferometer measurements at a distance of 9 m from the implemented pile—measurement diagram M4.1a (see Figure A1d). Time is plotted on the horizontal axis and the vertical axis represents the phase response in degrees.

Discrete frequency spectra (DFT) were generated using a two-second signal window, with the dominant amplitude representing the temporal center of the window. The Hanning window function was used and the number of samples was added so that the Fast Fourier Transform (FFT) algorithm could be used.

**Figure 8:**
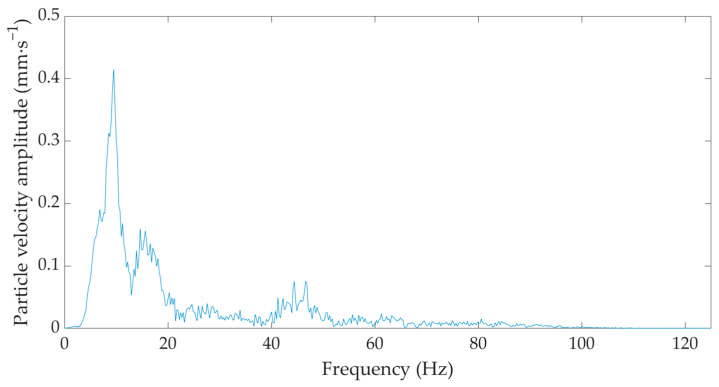
Frequency image of a single hammer strike during the implementation of a gravel pile from the seismic station BRS32—M4.1a measurement diagram.

**Figure 9:**
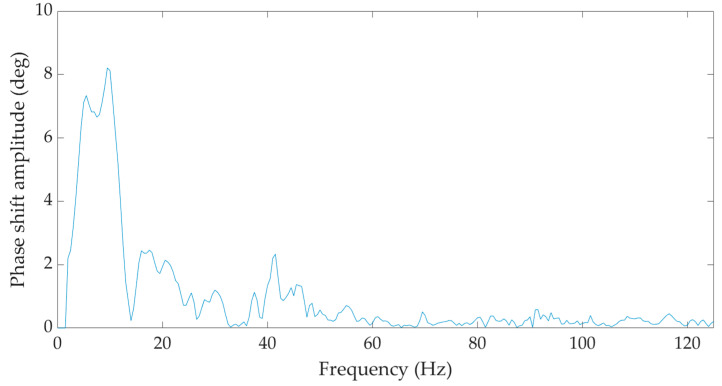
Frequency image of a single hammer strike during the implementation of a gravel pile from the fiber-optic system under development—M4.1a measurement diagram.

In the frequency domain, both the seismic station BRS32 and the optical equipment under development registered identical predominant peaks in the region of 5–10 Hz and 15–20 Hz for all measurements, as illustrated in Figure 8 and Figure 9, which show the frequency spectra corresponding to the time records taken from the measurements in situation 4—measurement diagram M4.1a (see Figure A1d).

The records taken from all registered gravel piles were very similar to each other, both in time and frequency domain. In the time domain, the individual phenomena had an identical peak length and peak character in a comparison between the BRS32 seismic station and the optical device under development.

With increasing distance from the source of the dynamic load, the vibration intensity, i.e., the maximum amplitude, only decreased according to the law of damping. In the frequency domain, both devices registered the same peak in the 5–10 Hz region. Significant differences in the data between measurements with identical equipment would likely indicate changes in local geology. Differences in the comparison between the electrical and optical devices would indicate a flaw in the design of the device under development. This way, the entire data set could be further processed and analyzed.

### Conversion of Measured Values between the Seismic Station and the Fiber-Optic System under Development

In total, 100 records from the BRS32 seismic station and 100 records from the fiber-optic system under development were processed in the time domain.

For subsequent data analysis and correlation, the procedure verified in the previous processing of data from laboratory measurements [1] was used. The least squares method was used to determine the regression function with the highest coefficient of determination R^2^, where the best fit was again a linear function. The results are summarized in Table 1, where the dependence shows a very high correlation coefficient R^2^. The maximum deviation from the measured values of the oscillation velocity amplitude was 2.287 mm·s^−1^, and the median deviation was 0.184.

**Figure 10:**
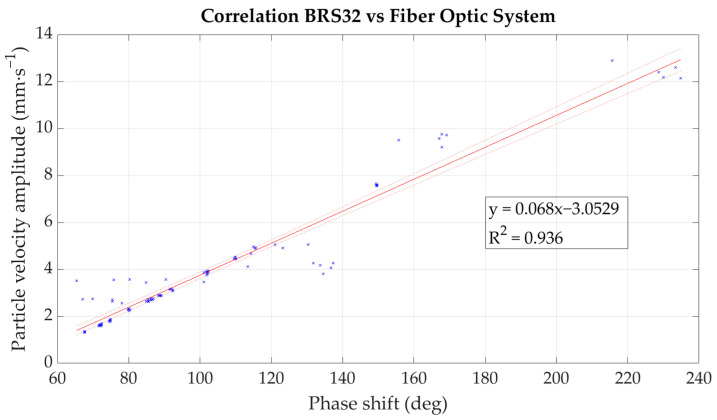
Mathematical dependence of the optical fiber system under development.

**Figure 11:**
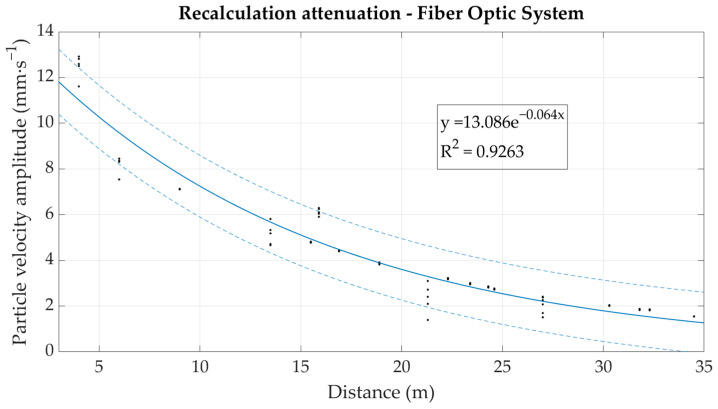
Graphical representation of the environmental attenuation based on the recalculated values measured by the optical interferometer.

**Figure 12:**
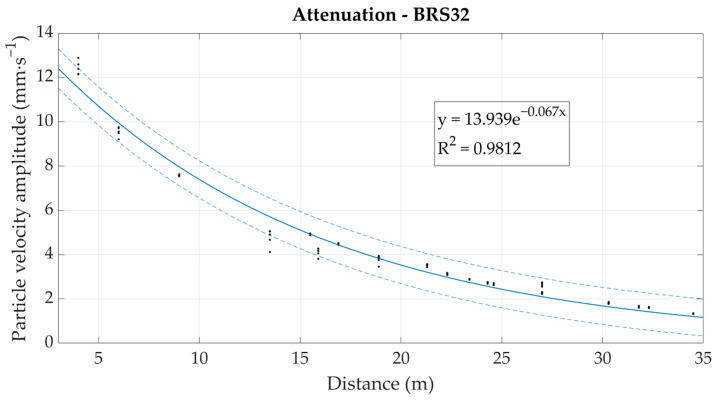
Graphical representation of the environmental attenuation from the values measured by seismic station BRS32.

Based on the correlation relationship in Figure 10, the measured values obtained from the fiber-optic system under development were converted to particle velocity in mm·s^−1^. These were subsequently plotted in the form of an attenuation curve (dependence of the maximum amplitude on the distance from the dynamic load source), see Figure 11. For comparison, this dependence was also plotted from the values obtained from seismic station BRS32, see Figure 12.

**Figure 13:**
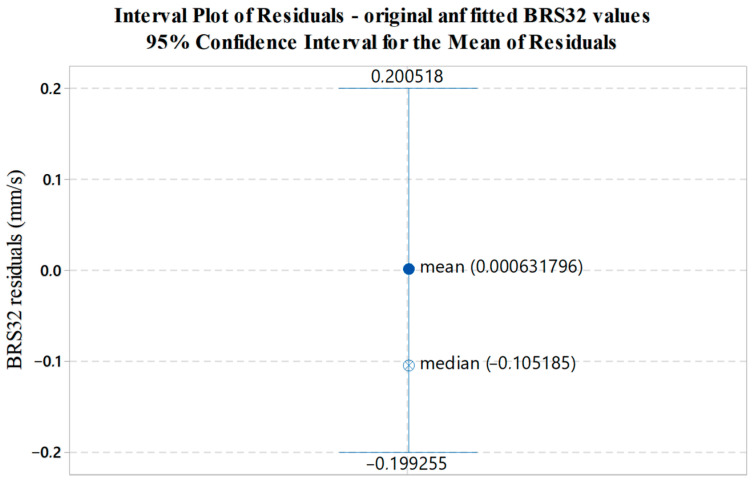
Graphical display of the variability of residuals (errors) between the measured and fitted BRS32 values with respect to data shown in Figure 10.

**Figure 14:**
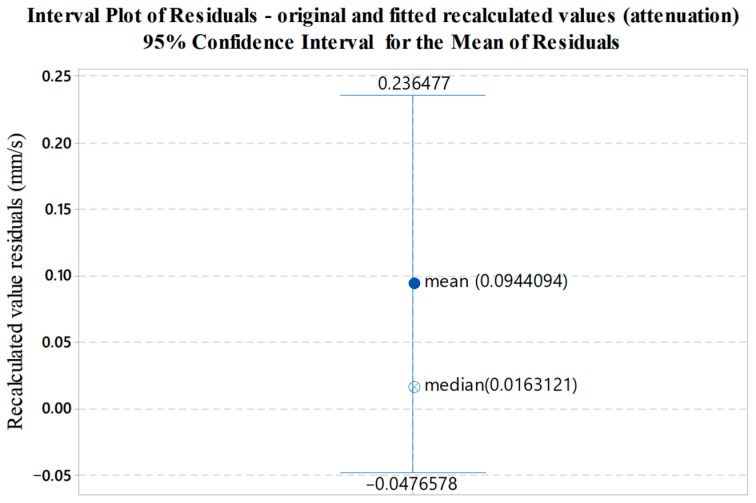
Graphical display of the variability of residuals (errors) between the measured and fitted recalculated values with respect to data shown in Figure 11.

**Figure 15:**
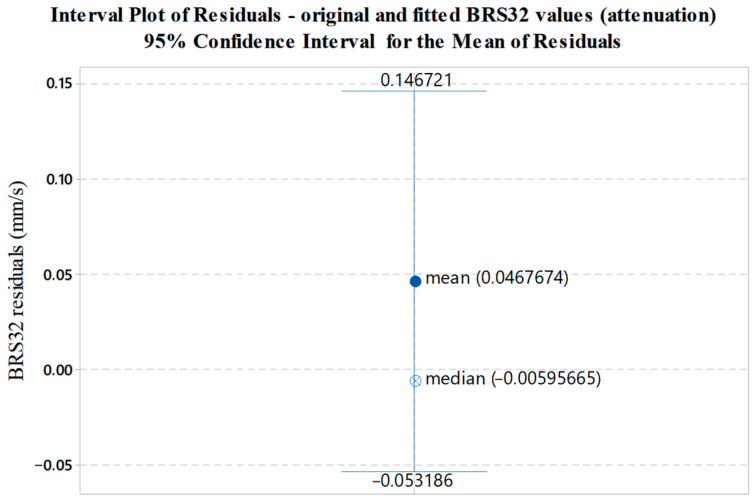
Graphical display of the variability of residuals (errors) between the measured and fitted BRS32 values with respect to data shown in Figure 12.

Interval bars presented in Figure 13, Figure 14 and Figure 15 illustrate the variability of residuals (errors) between the measured and fitted values. The mean value of residuals, corresponding median and 95% confidence interval for the mean are shown. 

## 6. Discussion

This article is a direct follow-up to a previous study [1] in which a correlation dependence between the values measured by conventional seismic monitoring instrumentation and an alternative sensing device based on a different physical principle was sought based on an experiment in laboratory conditions. A logical extension of this study was the deployment of the previously presented optical interferometer in field measurements. For further testing, the hammer strike was selected as the dynamic loading to act as a compaction agent in the implementation of gravel piles. Thus, once again, there is always one dynamic impact, similar to the calibrated impact in the case of the previous laboratory experiment.

Compared to the previous experiment, data collection was carried out over larger distances and a significantly higher range of distances from 5 to 35 m. In the original experiment, the substrate and bedrock were assumed to be completely homogeneous. In the case of the first in situ application, we can speak of an environment composed of quasi-homogeneous units, where a two-meter layer of fluvial gravels followed by a thick layer of clay with high plasticity was located under a one-meter layer of ballast. The entire measurement was carried out under normal climatic conditions (average temperature +15 °C, average humidity 75%, average atmospheric pressure 1025 hPa), as the influence of extreme climatic conditions has not yet been tested, and similarly a calibration test on a vibration table in a vacuum chamber is planned.

The most significant shortcoming of the presented interferometer design is its omnidirectionality, as standard seismic instrumentation works as a three-component seismometer that registers vibrations in three mutually perpendicular axes. The authors are developing a full-fledged three-component interferometric sensor, which is undergoing laboratory testing. However, as the results presented in this paper show, the correlation for the current interferometer design is also very high for the vertical direction at a value above 0.9, indicating that the chosen linear correlation relation, originally proposed for the results from the laboratory experiment, is correct. The maximum deviation from the measured values of the oscillation velocity amplitude this time was 2.287 mm·s^−1^, but the median deviation is 0.184, which is favorable considering the experimental nature and omnidirectionality of the interferometric sensor. In the frequency domain, there was maximum agreement between the registered peaks for all records.

The resulting attenuation curves of the rock environment plotted against the measured data are very similar in nature, and the correlation is again very favorable—above 0.9 in both cases, and the exponents in the attenuation equations show significant agreement with a deviation of 0.003 when compared to each other. Attenuation is determined this way in cases where it is required to predict the seismic loading of the surroundings under repeated seismic loading and in a rock environment that does not undergo significant change. The results show that fiber-optic sensors, in particular interferometric sensors, could also be used for this purpose.

Higher deviations for the attenuation environment were registered for closer distances, which is a consequence of the high sensitivity of the interferometers. This high sensitivity translates into high-frequency intensities in the case of the interferometer, which are then sensitive to the demodulation method used. It is therefore advisable not to place the sensor in the immediate vicinity of the vibration source or to ensure a sufficiently large bandwidth of the photodetector and the sampling rate of the measurement card. Therefore, the requirements for the AD converter are completely different from those of conventional measuring stations. While a high-precision (24–32 bit) ADC with a relatively small sampling rate is needed here, the interferometric sensor requires a particularly fast ADC with sufficient 16–24 bit accuracy.

## 7. Conclusions

The paper presented an interferometric sensor developed for the in situ monitoring of vibrations due to anthropogenic sources and using linear correlation dependence for the basic conversion of measured values to SI units. The fundamental innovation of the presented paper is mainly the application of technical seismicity in the field of geotechnical engineering and comparation with standard instrumentation for seismic monitoring—it was used as a reference instrument.

A total of 200 phenomena were evaluated in both the time and frequency domains, and the individual phenomena were similar in nature and character in both the frequency and time records. The correlation coefficient R^2^ was 0.936 and the average deviation from the measured values of the oscillation velocity was 0.0052 mm·s^−1^, which is very low.

The results of this experiment constitute part of the long-term research of the author’s team, the aim of which is a fully functional three-axis interferometric seismic sensor for use in geotechnical engineering. The conclusions from the in situ measurements show that the interferometric sensor (even if only one-axis) has the potential to replace standard seismic instrumentation even in field use, at least in an area where vibrations are generated by anthropogenic sources (harmonic vibrations of technological processes during geotechnical constructions, vibrations generated by traffic especially in cities and blasting in open pits near built-up areas or in excavated shallow tunnels), thus fully exploiting its many benefits.

## Figures and Tables

**Table 1:** Summary of results obtained from in situ comparative measurements.

	Correlation Equation	Correlation Coefficient R^2^
Seismic Station/Optical Interferometer	y = 0.068x − 3.0529	0.936
Optical Interferometer—attenuation	y = 13.086e^−0.064x^	0.926
Seismic station BRS32—attenuation	y = 13.939e^−0.067x^	0.981

## Data Availability

The data presented in this study are available on request from the corresponding author. The data are not publicly available due to privacy reasons.
